# Supplementary material for: Organic and inorganic sublattice coupling in two-dimensional lead halide perovskites
Source: Nat Commun. 2024 May 29;15:4562. doi: 10.1038/s41467-024-48707-1 (PMC11136976; doi:10.1038/s41467-024-48707-1)
Supplement: Supplementary file 1 — Supplementary Information [file 41467_2024_48707_MOESM1_ESM.pdf]

Supplementary Information

Supplementary Figures

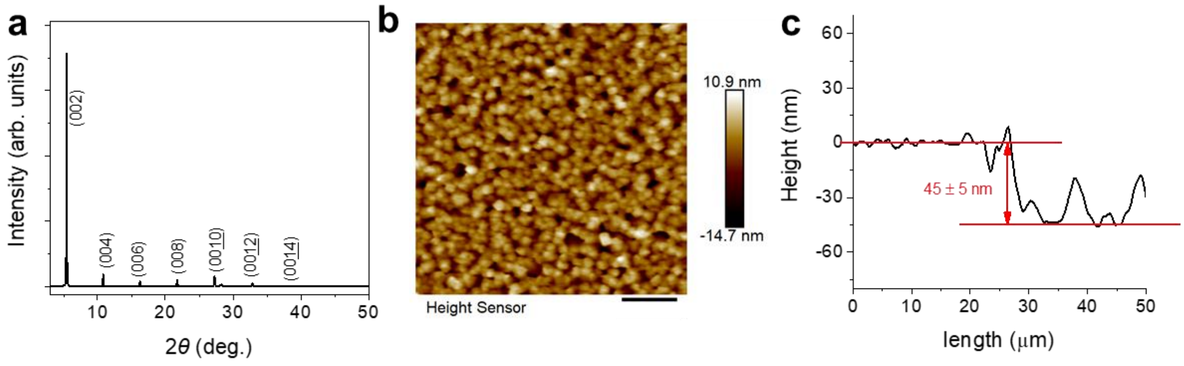

Supplementary Fig. 1| Basic characterization of the films. XRD pattern **a**, AFM morphology **b**, and step height profile **c** of $(PEA)_2PbI_4$ films. The scale bar in **b** is 8 μm. The XRD pattern shows that the film is preferentially oriented along the *c*-axis. The AFM measurements show that the film has a smooth surface and a thickness of 45 ± 5 nm.

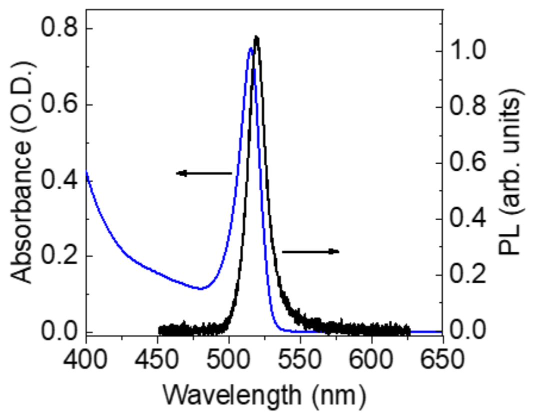

Supplementary Fig. 2| Optical characterization of the films. Linear absorption and PL spectra of $(PEA)_2PbI_4$ films at room temperature (RT). The linear absorption spectrum consists of continuum state transition and free exciton 1s transition centered at around 516 nm. The PL spectrum is dominated by the free exciton transition centered at 520 nm.

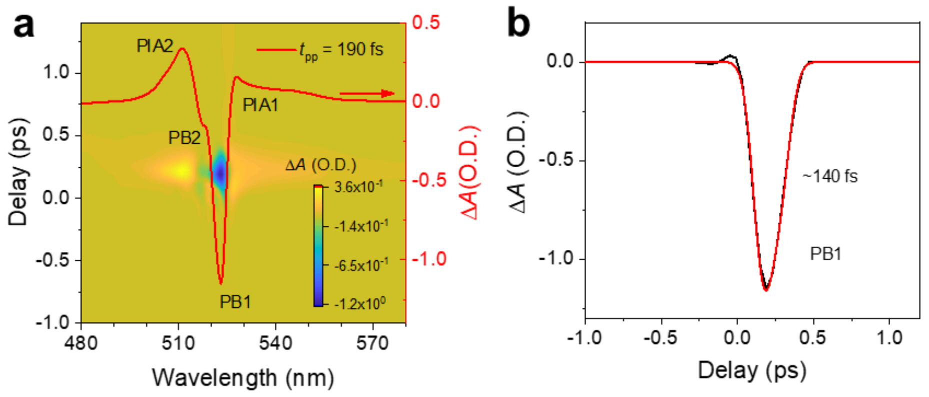

Supplementary Fig. 3| TA features near pump-probe time-zero. **a** 2D contour-plot of TA spectrum of $(PEA)_2PbI_4$ films at 77 K near the pump-probe overlap. The red curve is the TA spectrum at delay of 190 fs due to the optical Stark effect. **b** black curve: TA kinetics monitored at PB1. Red curve is the curve-fit using a gaussian function which yields a full-width-at-half-maximum (FWHM) of 140 fs.

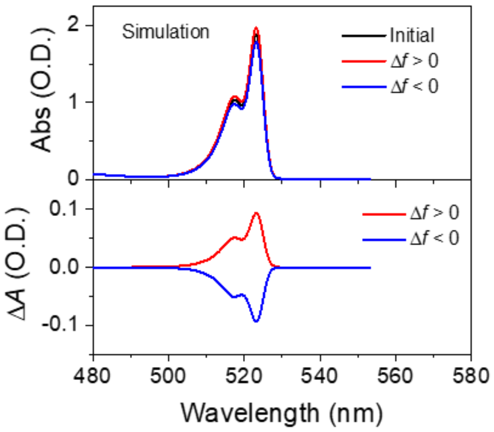

Supplementary Fig. 4| Absorption and TA spectra simulations. Simulated absorption spectra for the initial (black curve), with enhanced (red curve) and reduced (blue curve) oscillator strengths, and the resulting TA spectra when the oscillator strength is enhanced (red curve) or reduced (blue curve).

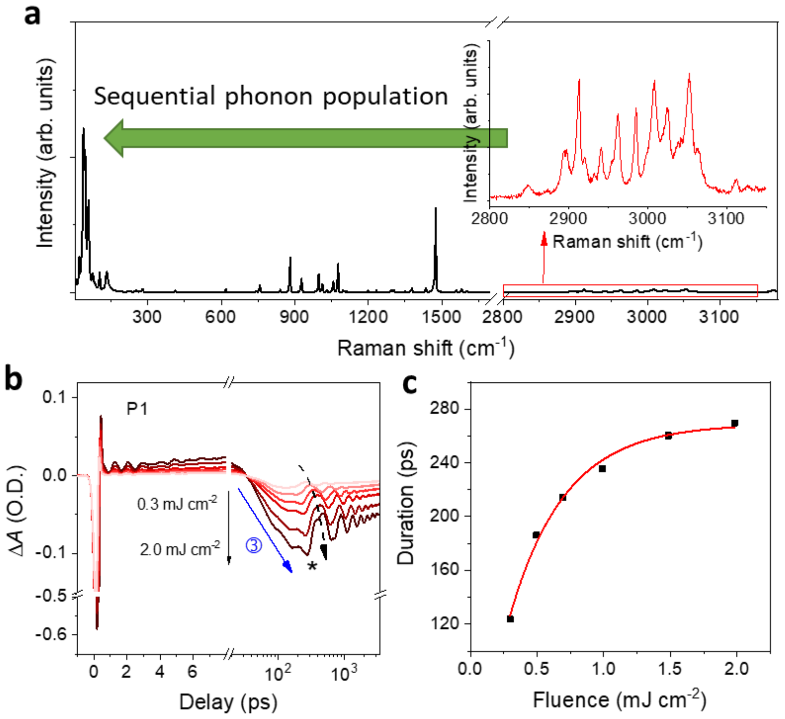

Supplementary Fig. 5| Observation of slow phonon-down conversion. a Raman spectrum of $(PEA)_2PbI_4$ at 77 K. Inset shows a zoom-in of the Raman spectrum that is dominated by the vibrational motion of PEA cation. **b** Fluence-dependent TA kinetics of $(PEA)_2PbI_4$ films monitored at P1 with pump at 3.3μm. **c** Duration of regime ③ as a function of pump fluence (scatters) and the corresponding curve-fit (red curve) using $y = y_0 + A\exp\left(-\frac{I}{I_0}\right)$, where A, y_0 and I_0 are constants.

As reported in previous works[1,2], the low-frequency phonon modes (< ~150 cm^{-1}) are dominated by the vibrational motion of the PbI_6 octahedra with mode located at ~135 cm^{-1} exhibiting mixed contributions from PEA cations and PbI_6 octahedra whereas the high-frequency phonon modes (> 200 cm^{-1}) are governed by the vibrational motion of PEA cations. The vibrational coupling between these two sublattices is thus relatively weak.

Note that in Supplementary Fig. 5a, the phonon down-conversion process involves two successive processes: intramolecular energy redistribution within the PEA cation followed by the heat transfer from organic PEA cation to inorganic PbI_6 octahedra through anharmonic coupling.[3,4] For the former, it involves sequential energy down-conversion through all the intermediate phonon modes starting from the excited high-energy phonon mode (*i.e.*, N-H stretching motion) to the lowest-lying phonon modes. The duration of this process is in general longer than the lifetime of activated phonon modes. For the latter, it involves the heat transfer from the phonon modes of organic cation to those of inorganic octahedra which is governed by the overlapping vibrational density of states and the coupling between the organic and inorganic sublattices.[3,5] Nevertheless, the large mass difference which results

in a large energy difference of the phonon modes as well as their relatively weak coupling though hydrogen bonding and electrostatic interaction between these two sublattice lead to a significant weak mechanical coupling, thereby slow heat transfer. This inefficient mechanical coupling results in slow heat transfer which is evident from the increase of duration time of regime ③ with pump fluence.

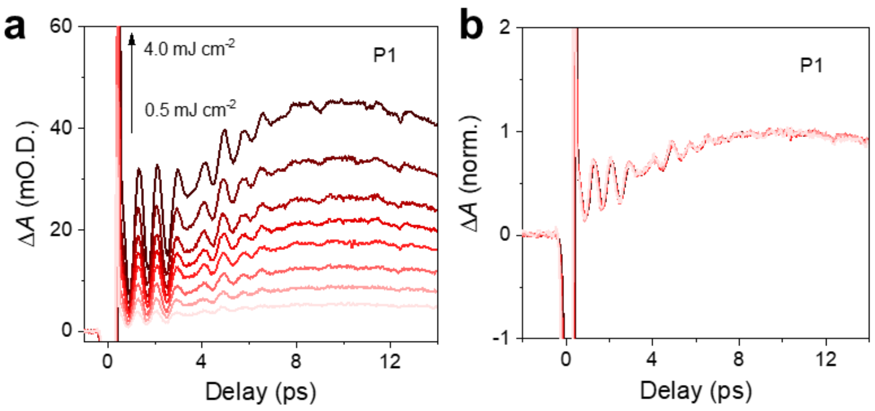

Supplementary Fig. 6| TA kinetics in the first ~15 ps. a Fluence-dependent TA kinetics in the first ~15 ps of $(PEA)_2PbI_4$ films monitored at P1 with pump at 3.3μm. **b** Normalized fluence-dependent TA kinetics monitored at P1.

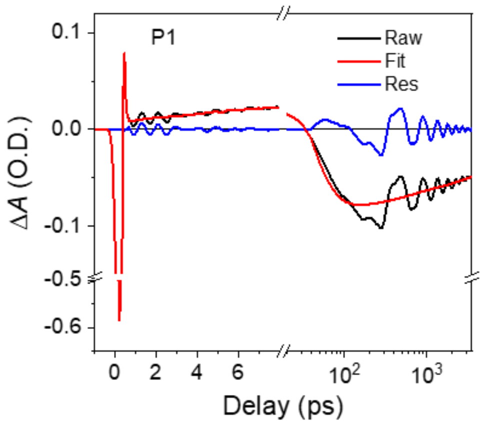

Supplementary Fig. 7| TA kinetics and curve fittings. TA kinetics (black curve), the curve-fit (red curve) and the fitting residual (blue curve) monitored at P1 of $(PEA)_2PbI_4$ films pumped with a MIR laser pulse of 3.3 μm with a fluence of 2 mJ cm^{-2} at 77 K.

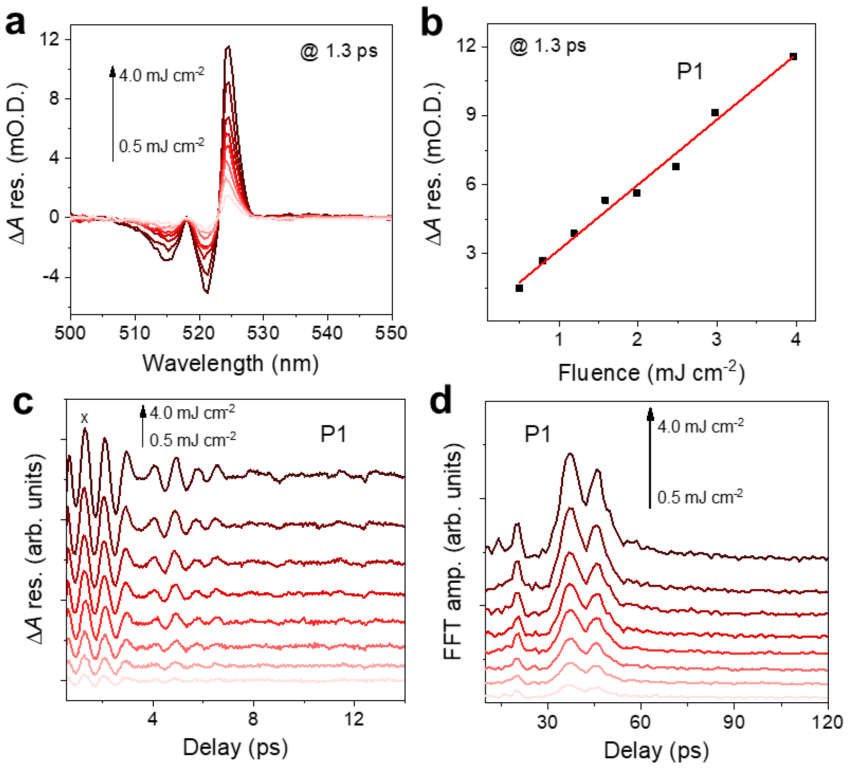

Supplementary Fig. 8| Fluence-dependent oscillation kinetics. **a** Pump fluence-dependent phonon modulated TA spectrum of $(PEA)_2PbI_4$ films pumped at 3.3 μm at delay of 1.3 ps. **b** Phonon induced ΔA amplitude monitored at P1 as a function of pump fluence. The filled scatters and the red line are the experimental data and the linear fit, respectively. **c** Pump fluence-dependent TA oscillation kinetics monitored at P1. **d** Pump fluence-dependent FFT amplitude spectrum.

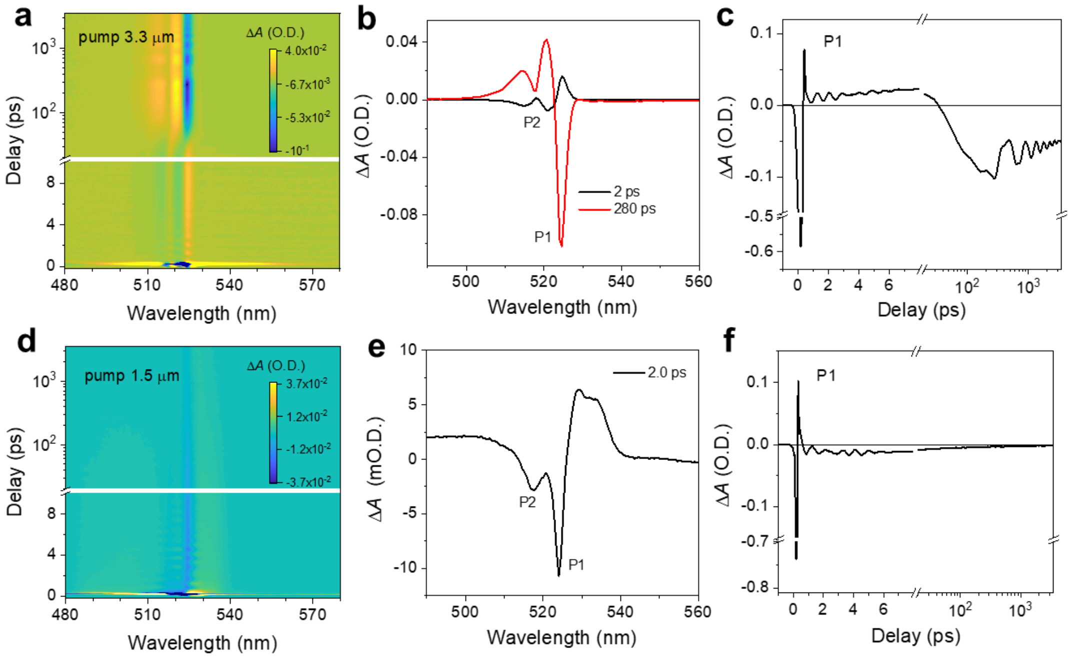

Supplementary Fig. 9| TA features for resonant and off-resonant excitations. comparison of 2D contour-plot of TA spectrum, representative TA spectrum at different delay times and TA kinetics monitored at P1 of (PEA)2PbI4 films obtained with resonant pump using a MIR laser pulse at 3.3 μm (**a**, **b**, and **c**) and with off-resonant pump using an IR laser pulse at 1.5 μm (**d**, **e**, and **f**). Note that **a**, **b** and **c** are replots of Figure 1 of the main text.

Supplementary Fig. 9 shows a comparison of the distinct TA spectra generated by resonant pump of PEA cations using a MIR laser pulse versus that by off-resonant pump using an IR laser pulse at 1.5 μm. Apart from the same COPs-induced oscillation component, the former features a positive ΔA (peak P1) due to reduction of exciton energy and is followed by a negative ΔA at longer times because of the increase of exciton energy arising from thermal lattice expansion. Conversely, the latter is characterized by a negative ΔA due to band-filling of the ground-state absorption owing to multiphoton absorption.

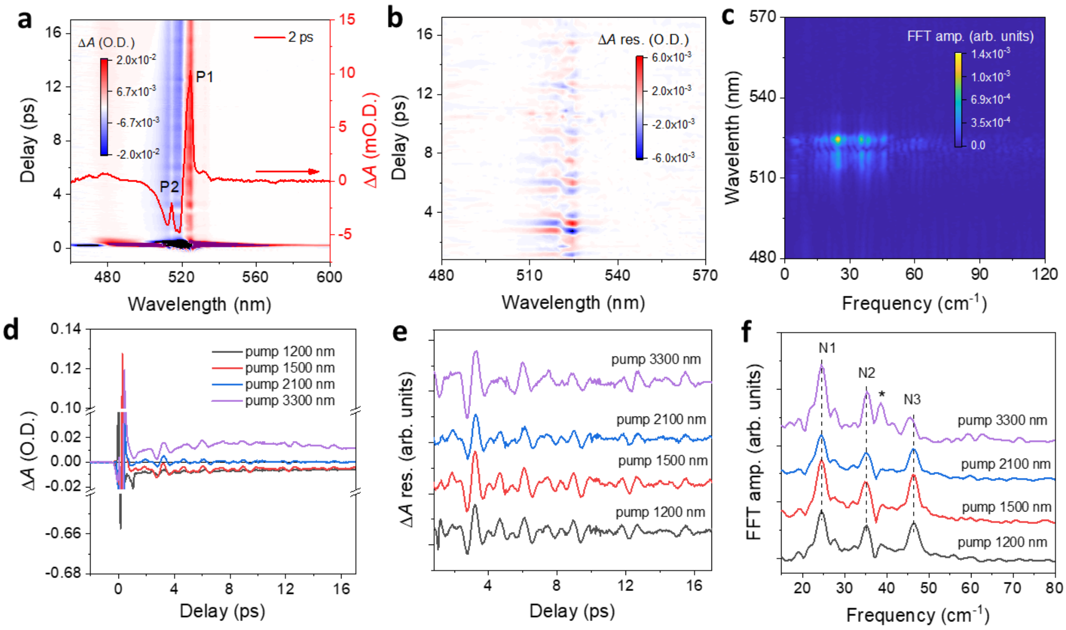

Supplementary Fig. 10| Observation of electron coupling between two sublattices in $(HA)_2PbI_4$. a 2D contour-plot of TA spectrum of $(HA)_2PbI_4$ films pumped at 3.3 μm with a fluence of 1.2 mJ cm^{-2}. 2D contour-plot of the phonon induced beating map in the time-domain **b** and frequency-domain **c**. Pump energy-dependent TA kinetics **d**, the phonon induced oscillation kinetics **e**, and the corresponding calculated FFT spectra **f** monitored at P1. The dashed lines in **f** are the guidelines to the eye indicating the peak positions. As seen, slight blue-shifts of N1 and N2 modes, and red-shift of N3 are present for MIR pump.

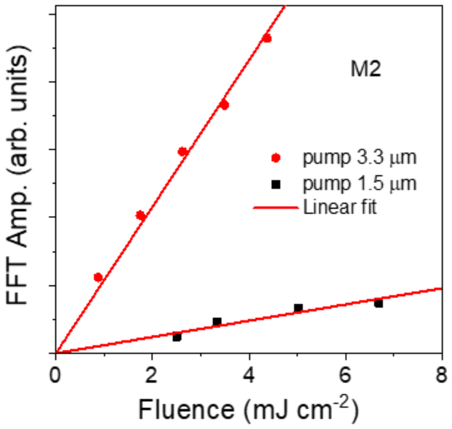

Supplementary Fig. 11| Fluence-dependent oscillation amplitude. FFT amplitude of M2 mode as a function of pump fluence for off-resonant IR pump at 1.5 μm (black squares) and resonant MIR pump at 3.3 μm (red circles). The red lines are linear fits.

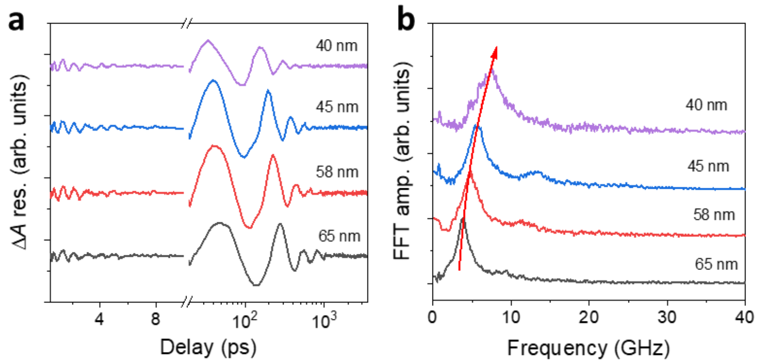

Supplementary Fig. 12| Presence of COPs and CLAPs in $(PEA)_2PbI_4$ films with different thicknesses. Thickness dependence of oscillation kinetics in $(PEA)_2PbI_4$ films which consists of contributions from COPs in the first 20 ps that is independent of thickness **a** and CLAPs in the later several ns with its oscillation frequency decreases with thickness **b**. The samples were resonantly excited at 3.3 µm with a fluence of 0.4 mJ cm^{-2}. The measurements were conducted at 77 K.

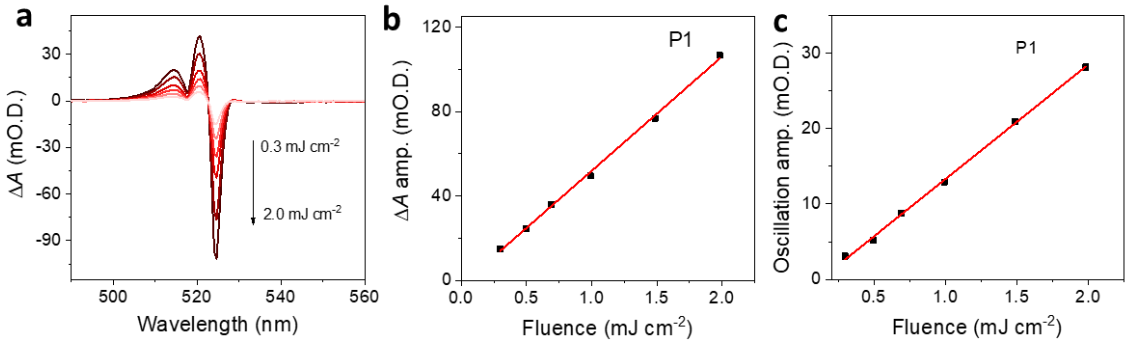

Supplementary Fig. 13| TA features during phonon down-conversion process. a Fluence-dependent phonon down-conversion process-induced transient TA spectrum of $(PEA)_2PbI_4$ films. The data is extracted when the ΔA amplitude at P1 is maximum. **b** The induced transient ΔA amplitude (filled scatters) monitored at P1 as a function of pump fluence and the linear fit (red line). **c** CLAPs-induced transient ΔA oscillation amplitude (filled scatters) as a function of pump fluence and the linear fit (red line).

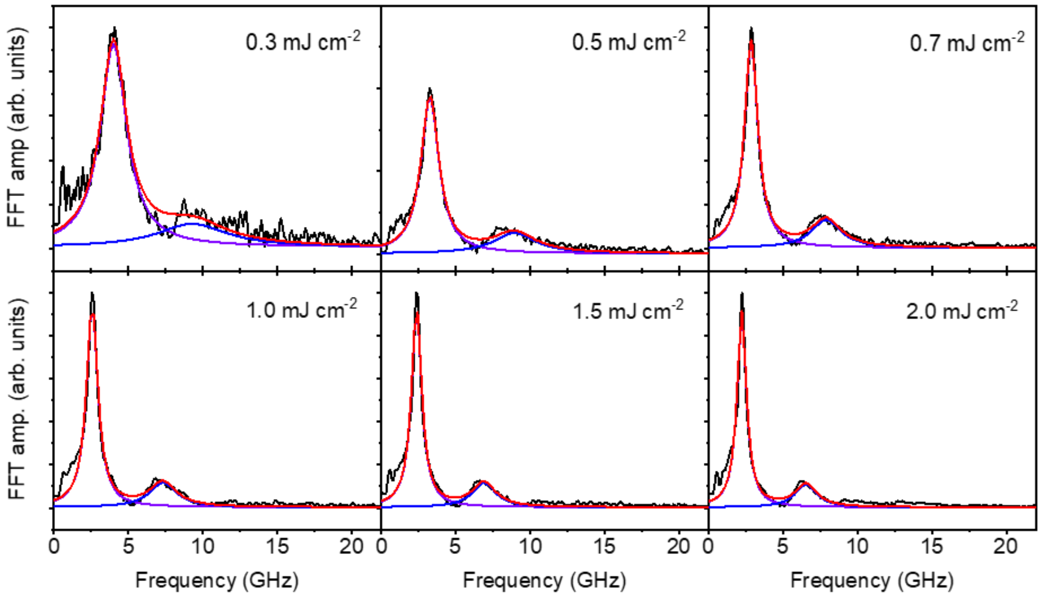

Supplementary Fig. 14| Fluence-dependent CLAPs features. FFT spectra and the curve-fits using a sum of two Lorentz functions of the oscillation kinetics induced by CLAPs for different pump fluences of $(PEA)_2PbI_4$ films. The black, red, green and blue curves are the experimental data, the contributions from the low and high-frequency components and the sum of these two components, respectively. As seen, the asymmetry from the low-frequency component is negligible.

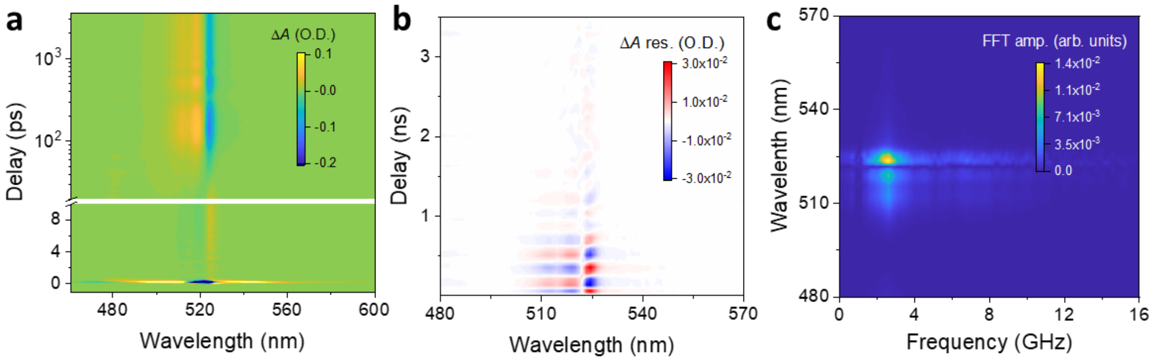

Supplementary Fig. 15| Presence of COPs in $(HA)_2PbI_4$ films with MIR pump. 2D contour-plot of TA spectrum of $(HA)_2PbI_4$ films **a** pumped at 3.3 μm with a fluence of 0.6 mJ cm^{-2}. 2D contour-plot of the CLAPs induced beating map in the time-domain **b** and frequency-domain **c**.

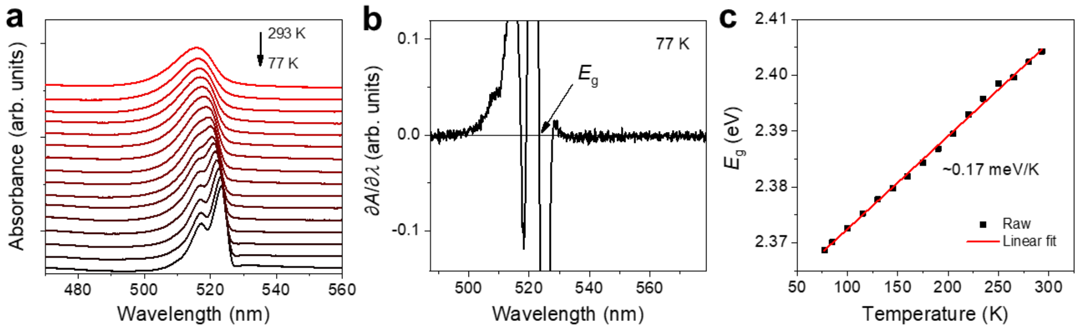

Supplementary Fig. 16| Temperature-dependent optical bandgap. a Temperature-dependent linear absorption spectrum of $(PEA)_2PbI_4$ films. **b** $\boldsymbol{\partial A/\partial \lambda}$ as a function of wavelength for $(PEA)_2PbI_4$ films at 77 K. The crossing point indicated by the arrow corresponds to the optical bandgap. **c** Extracted optical bandgap (filled scatters) as a function of temperature and the linear fit (red line).

The temperature dependence of the bandgap under quasi-harmonic approximation consists of contributions from thermal expansion and electron-phonon interactions:[6]

$$\frac{\partial E_{\mathrm{g}}}{\partial T} = \frac{\partial E_{\mathrm{g}}}{\partial V}\frac{\partial V}{\partial T} + \sum_{j,\vec{q}} \frac{\partial E_{\mathrm{g}}}{\partial n_{j,\vec{q}}}\left(n_{j,\vec{q}} + \frac{1}{2}\right) \tag{1}$$

where $n_{j,\vec{q}}$ is the number of phonons at j branch with wave vector $\vec{q}$, V is the volume. Normally, $\frac{\partial E_{\mathrm{g}}}{\partial V}$ is determined by the bonding feature of the atomic orbitals and is thus a constant that is weakly dependent on temperature in a single phase. The second term of Eq. (1) refers to the phonon contribution that varies with temperature because of the different phonon occupation number. By assuming that the lattice constant shows a linear temperature dependence, Eq. (1) is usually simplified as:[7]

$$E_{\mathrm{g}}(T) = E_{\mathrm{g}}(0) + AT + \sum_{i} B_i\left(n_i + \frac{1}{2}\right) \tag{2}$$

where i refers to the ith phonon contribution with occupation number n_i and contribution factor B_i.

We first determine the $(PEA)_2PbI_4$ film's optical bandgap (free exciton) which is the low-energy peak position in the absorption spectrum. This peak position corresponds to the crossing point in the $\partial A/\partial \lambda$ spectrum, as shown in Supplementary Fig. 16b. The extracted optical bandgap as a function of temperature is displayed in Supplementary Fig. 16c. The optical bandgap exhibits a linear dependence on the temperature, indicating that the electron-phonon coupling only plays a negligible role. Linear fitting of the optical bandgap with temperature yields the value of A = 0.17 meV/K.

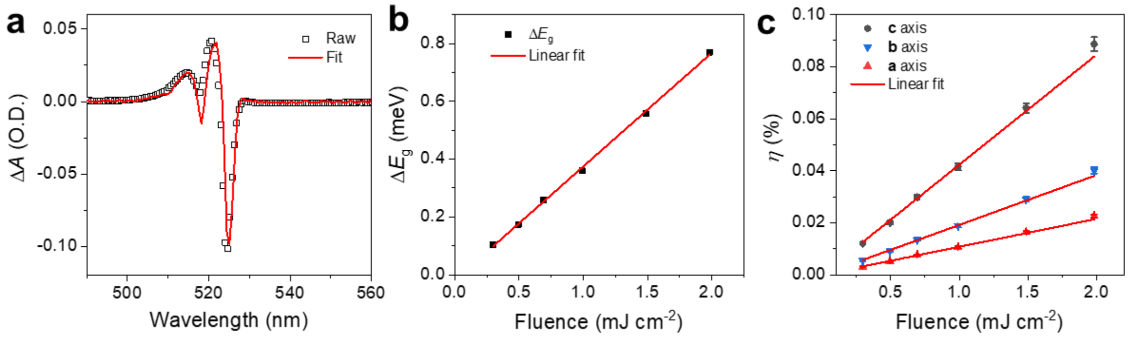

Supplementary Fig. 17| Tensile strain-induced increase of optical bandgap. a Representative TA spectrum and curve-fit of $(PEA)_2PbI_4$ films at delay of 280 ps. The sample was pumped at 3.3 μm with a fluence of 2 mJ cm^{-2}. **b** Extracted bandgap shift as a function of pump fluence (black scatters) and the linear fit. **c** Calculated tensile strain (filled scatters) and the linear-fit (red lines) along different axis of the unit cell. The error bars in **b** come from curve-fit in **a**.

To estimate the amplitude of the induced tensile strain $\eta = \Delta l/l$, we first approximate the induced ΔA by the temperature increase ΔT due to the phonon down-conversion process to the first-order as: $\Delta A = \frac{\partial A}{\partial E_g}\frac{\partial E_g}{\partial T}\Delta T$. The tensile strain η is then estimated as $\eta = \beta\Delta T$, where $\beta = \frac{1}{L}\frac{dL}{dT}$ is the linear thermal expansion coefficient.[8] As shown, the estimated strains along all the axis increase linearly with pump fluence. However, the out-of-plane (along **c**-axis) strain is much larger than that of the in-plane strain (inorganic PbI_6 plane). This can be attributed to a much weaker van der Waals bonding between the organic spacers as compared to that of stronger covalent bonding between Pb and I atoms such that the *c*-axis is easier to expand or contract with temperature variation compared with other two axes.

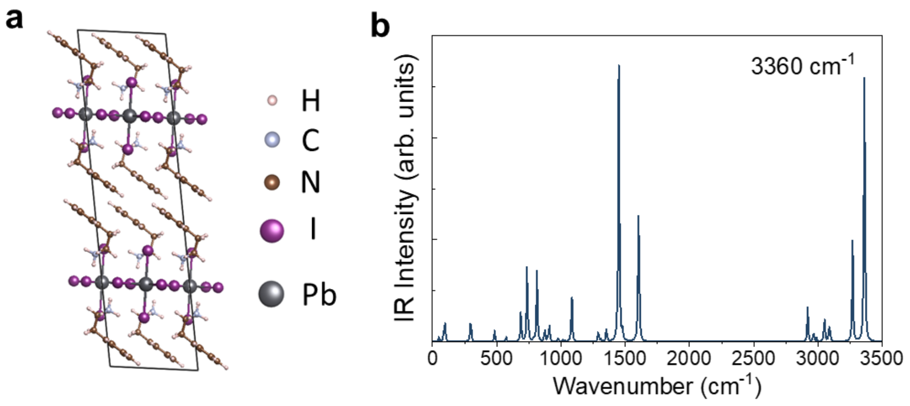

Supplementary Fig. 18| Optimized structure in DFT calculations. a Optimized crystal structure of $(PEA)_2PbI_4$. **b** Calculated IR spectra of PEA^+ molecule at B3LYP/6-31G(d,p) level (scaling factor = 0.961).

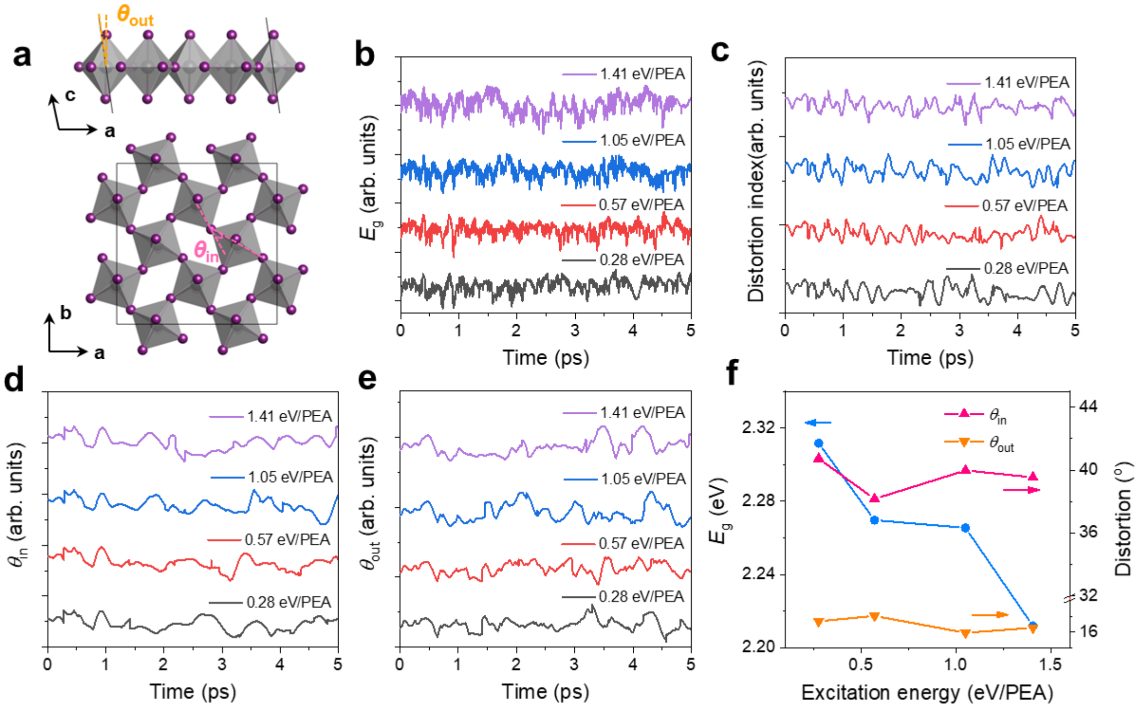

Supplementary Fig. 19| Structure parameters simulations. a Schematic of the out-of-plane distortion θ_{out} and in-plane distortion θ_{in} of the inorganic octahedra. The plane is defined by three adjacent Pb atoms that are parallel to the inorganic layer. The AIMD simulated kinetics for the bandgap E_g **b**, distortion index **c**, θ_{in} **d** and θ_{out} **e** after exciting N−H stretching motion with several excitation energies. **f** Average bandgap E_g, θ_{in} and θ_{out} as a function of excitation energy.

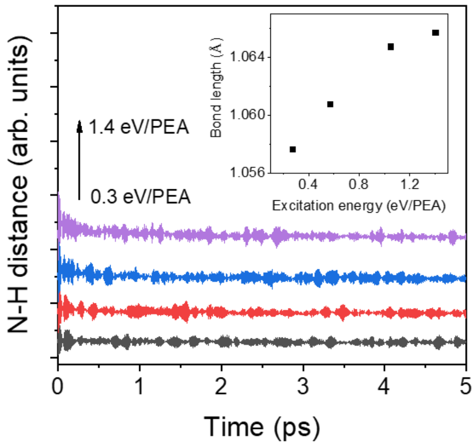

Supplementary Fig. 20| N−H bond length simulations. Simulated ensemble averaged N−H bond length kinetics after exciting N−H stretching motion with different excitation energies. Inset is the time-averaged N−H bond length as a function of excitation energy.

Supplementary Tables

Supplementary Table 1| Fitting details. Fitted parameters of Fig. 1c of the main text using Eq. (7). See Supplementary Note 1.

	P1	P2
A_0 (mOD)	40 ± 2	
L (x10^{-4})	7.0 ± 1.3	
C (mOD)	48.4 ± 0.3	28.0 ± 0.3
ω_0 (meV)	2363.5 ± 0.8	2391.8 ± 0.2
E_b (meV)	215.9 ± 8.4	80.0 ± 24.4
γ_X (meV)	8.8 ± 0.1	10.2 ± 0.4
γ_C (meV)	42 ± 17	300 ± 69
η (x10^{-3})	12.4 ± 0.1	20.6 ± 0.4
s	1.2 ± 0.1	2.0 ± 0.1

Supplementary Table 2| Fitting details. Fitted parameters of Fig. 2b of the main text.

	P1	P2
$\Delta\omega_0$ (meV)	(-2.8 ± 0.1)x10^{-2}	(-2.8 ± 0.2) x10^{-2}
$\Delta\gamma_X$ (meV)	(-8.3± 1.2)x10^{-3}	(-1.7± 0.3)x10^{-2}
$\Delta\gamma_C$ (meV)	(6.6 ± 1.2)x10^{-2}	(-4.7 ±0.5) x10^{-1}

Supplementary Notes

Supplementary Note 1| Low-temperature absorption model

The fitting procedure for the low-temperature absorption spectrum of $(PEA)_2PbI_4$ films can be referred to our previous work.[9] Briefly, the spectrum is fitted using a quantum-well absorption model which consists of discrete and continuum state transitions that are given by:

$$\alpha(E) = \sum_{n=1}^{\infty} \frac{2}{(n-q)^3} \delta\left(\frac{E}{R} + \frac{1}{(n-q)^2}\right) + \theta\left(\frac{E}{R}\right) \tag{3}$$

Here, R is the exciton Rydberg, $q = 1 - \sqrt{R/E_\mathrm{b}}$ is a parameter to quantify the dimensionality of the system, E_b is the exciton binding energy, and θ is the step function. Taking into account the linewidth broadening due to exciton localization induced by potential energy fluctuation arising from the structural or compositional disorder[10,11] or polaronic effect[12,13] stemming from the strong exciton-phonon coupling. The absorption spectrum is thus expressed as:[10]

$$A(\hbar\omega) = A_\mathrm{Dis} + A_\mathrm{Con} \tag{4}$$

The lineshape of the discrete state transition $\mathrm{A_{Dis}}$ is given by:

$$A_\mathrm{Dis} = \frac{1}{2\eta}\left[1 + \mathrm{erf}\left(\frac{\Delta}{\gamma_\mathrm{X}} - \frac{\gamma_\mathrm{X}}{2\eta}\right)\right] \exp\left(\frac{{\gamma_\mathrm{X}}^2}{4\eta^2} - \frac{\Delta}{\eta}\right) \tag{5}$$

Here, $\Delta = \omega - \omega_0$, ω_0 is the average exciton energy, γ_X is the linewidth for discrete state transition, erf(x) is the error function and η is a parameter describing the asymmetric linewidth broadening. The lineshape of the continuum state transition A_Con is expressed as:

$$A_\mathrm{Con} = \frac{s}{2}\left[1 + \mathrm{erf}\left(\frac{\Delta - E_\mathrm{b}}{\gamma_\mathrm{C}}\right)\right] \tag{6}$$

where γ_C and s are respectively the linewidth and step height of the continuum state transition. Here, to reproduce the low-temperature linear absorption spectrum, we phenomenologically consider two exciton states. Considering the Rayleigh scattering background, the absorption spectrum is then fitted with:

$$A(E) = C_1 A_1(E) + C_2 A_2(E) + A_\mathrm{BG} \tag{7}$$

where C_1 and C_2 are prefactors, $A_{\mathrm{BG}} = A_0 - \log(1 - L\omega^4)$, where C is a constant and L is a constant of proportionality.

Supplementary Note 2| Lattice dynamics for mid-infrared pump

The lattice dynamics after ultrashort pulse excitation can in general be described by the classical driven damped harmonic oscillator model, which is given by:

$$\frac{d^2Q}{dt^2} + 2\beta\frac{dQ}{dt} + \Omega_0^2 Q = \frac{F}{\mu} \quad (8)$$

where Q is the lattice displacement, β is the damping constant, μ is the reduced lattice mass, Ω_0 is the natural frequency of the oscillator, and F is the driving force due to the dipolar interaction between the phonon mode and the electric field of the laser pulse. Within the assumptions of two-band process dominated Raman tensor and varnishing decay rate of the coupled carriers, the driving force for opaque samples consists of contributions from both virtual and real excitations:[14]

$$F(\Omega) = -C\Xi\left[\frac{\partial\epsilon_{\mathrm{R}}}{\partial\omega} + 2i\frac{\varepsilon_{\mathrm{Im}}}{\Omega}\right] I(\Omega) \quad (9)$$

where C is a prefactor, ε_{R} and $\varepsilon_{\mathrm{Im}}$ are, respectively, the real and imaginary parts of the dielectric constant, Ξ is the deformation potential constant. And $I(\Omega) = \int_{-\infty}^{+\infty} e^{i\Omega t}|E(t)|^2 dt$, where the electric field of the laser pulse $E = Ae^{-t^2/2\tau_{\mathrm{L}}^2}\cos\omega_{\mathrm{L}}t$, A is the electric field amplitude, τ_{L} is the laser pulse duration, ω_{L} is the central pulse frequency. The first term in the parentheses of Eq. (9) corresponds to the virtual excitation which is the case for the ground-state ISRS whereas the second term in the parentheses of Eq. (9) refers to the real excitation arising from single- or multiple-photon excitation. The solution to Eq. (9) has the expression:

$$Q = Q_0 e^{-\beta t}\cos(\Omega_1 t + \phi) \quad (10)$$

where $Q_0 \approx \frac{\sqrt{2\pi}}{\Omega_1} C\Xi I \sqrt{\left(\frac{\partial \epsilon_\mathrm{R}}{\partial \omega}\right)^2 + 4\left(\frac{\epsilon_\mathrm{Im}}{\Omega_1}\right)^2}$ is the lattice displacement amplitude, $\Omega_1 = \sqrt{\Omega_0^2 - \beta^2}$ and $\phi = \arctan\left(\frac{\Omega_1}{2\epsilon_\mathrm{Im}} \frac{\partial \epsilon_\mathrm{R}}{\partial \omega} - \frac{\beta}{\Omega_1}\right)$ is the phase constant which is related to the ratio of the contribution of virtual excitation to that of real excitation.

Supplementary Note 3| Estimation of photoexcited PEA cation

We note that our simulated result (~10 meV) is nearly 2 orders of magnitude larger compared to the experimental results (~0.1 meV). This is likely because we excite all the PEA cation in our simulations while only a small fraction of PEA cation is excited in the sample. To verify this, we first estimate the number of excited PEA in the sample using:

$$n = \frac{P\alpha}{hv} \tag{11}$$

where P is the pump power, α is the IR absorption coefficient, and hv is the pump energy. For typical pump power of ~1 mJ cm^{-3}, α of ~ 2×10^{3} cm^{-3}, n is estimated as 3.3×10^{20} cm^{-3}.

And the number of PEA cation N in 1 cm^{3} is calculated using:

$$N = 8\frac{\rho}{M} N_\mathrm{A} \tag{12}$$

where ρ is the mass density (~2.55 g cm^{-3}), M is the molar mass density (959.19 kg mol^{-1}), N_A is the Avogadro's number. Here, N is estimated as ~1.3×10^{22}. The faction of excited PEA cation is then estimated as n/N ~2.6×10^{-2}. Assuming a linear relationship between the optical bandgap and pump power which holds for our case based on our experimental results (Fig. 2c of the main text), the estimated reduced optical bandgap is thus reasonable.

Supplementary References

1 Thouin, F. *et al.* Phonon coherences reveal the polaronic character of excitons in two-dimensional lead halide perovskites. *Nat. Mater.* **18**, 349-356 (2019).

2 Thouin, F. *et al.* Stable biexcitons in two-dimensional metal-halide perovskites with strong dynamic lattice disorder. *Phys. Rev. Mater.* **2**, 034001 (2018).

3 Guo, P. *et al.* Infrared-pump electronic-probe of methylammonium lead iodide reveals electronically decoupled organic and inorganic sublattices. *Nat. Commun.* **10**, 1-8 (2019).

4 Diroll, B. T., Kamysbayev, V., Coropceanu, I., Talapin, D. V. & Schaller, R. D. Heat-driven acoustic phonons in lamellar nanoplatelet assemblies. *Nanoscale* **12**, 9661-9668 (2020).

5 Ong, W.-L., Rupich, S. M., Talapin, D. V., McGaughey, A. J. & Malen, J. A. Surface chemistry mediates thermal transport in three-dimensional nanocrystal arrays. *Nat. Mater.* **12**, 410-415 (2013).

6 Yu, C. *et al.* Temperature dependence of the band gap of perovskite semiconductor compound $CsSnI_3$. *J. Appl. Phys.* **110**, 063526 (2011).

7 Bhosale, J. *et al.* Temperature dependence of band gaps in semiconductors: Electron-phonon interaction. *Phys. Rev. B* **86**, 195208 (2012).

8 Wang, H. *et al.* Interfacial residual stress relaxation in perovskite solar cells with improved stability. *Adv. Mater.* **31**, 1904408 (2019).

9 Fu, J. *et al.* Electronic states modulation by coherent optical phonons in 2D halide perovskites. *Adv. Mater.* **33**, 2006233 (2021).

10 Schnabel, R. *et al.* Influence of exciton localization on recombination line shapes: $InxGa_{1-x}As$/GaAs quantum wells as a model. *Phys. Rev. B* **46**, 9873 (1992).

11 Leosson, K., Jensen, J. R., Langbein, W. & Hvam, J. M. Exciton localization and interface roughness in growth-interrupted GaAs/AlAs quantum wells. *Phys. Rev. B* **61**, 10322 (2000).

12 Neutzner, S. *et al.* Exciton-polaron spectral structures in two-dimensional hybrid lead-halide perovskites. *Phys. Rev. Mater.* **2**, 064605 (2018).

13 Thouin, F. *et al.* Stable biexcitons in two-dimensional metal-halide perovskites with strong dynamic lattice disorder. *Phys. Rev. Mater.* **2**, 034001 (2018).

14 Stevens, T., Kuhl, J. & Merlin, R. Coherent phonon generation and the two stimulated Raman tensors. *Phys. Rev. B* **65**, 144304 (2002).
